# Hysteria

**Published:** 1889-10

**Authors:** James Wright Putnam

**Affiliations:** Clinical Lecturer on Nervous Diseases in the Medical Department, University of Buffalo; 388 Franklin Street


### HYSTERIA.[1]

By JAMES WRIGHT PUTNAM, M. D.,

Clinical Lecturer on Nervous Diseases in the Medical Department, University of Buffalo.

It would seem that an apology were necessary before presenting a paper on Hysteria before a pathological society, for the science of to-day has repudiated the pathology of the past, and has offered nothing to replace it.

---

1. Read before the Buffalo Pathological Society, September 20, 1889.

Because of the importance and frequency of the hysterical condition, I wish to present some facts relating to its diagnosis: In the first place we must say that nineteen-twentieths of the cases are found in the female sex, and that the graver forms of hysteria occur in both sexes, in the old and young.

There are certain points in aetiology that must be considered. Heredity in hysterics to parents with nervous disease, or brain disease, may be traced in twenty-five per cent., while thirty-three per cent. of the children born of hysterical mothers are hysterical.

Climate does not appear to influence hysteria, neither does social position, for, like the gout, it attacks the poor and the rich. City life predisposes to it more than country life. The mode of education and the mode of life and choice of books have their influence. Before Briquet's masterly study of hysteria, it was the ordinary belief that continence in women was a powerful factor, as illustrated by the number of cases occurring in convents.

The fact, on more careful study, shows that the nuns who are members of the active charitable orders which require them to give their minds and bodies certain healthy employment, are relatively free from hysteria, but that in the mystical and contemplative orders, hysteria was frequent; while, on the other hand, in Paris hysteria was found to be present in a large percentage of prostitutes by Briquet. And on his authority we base the statement that continence does not cause hysteria; we may look for this disease as a complication to organic disease, and to such general conditions as anemia, sclorosis and rheumatism. After profound mental emotions, as fear and sorrow; after accidents, causing injuries, we may also see hysteria develop, also after great fatigue.

A few rules must be borne in mind when looking for this condition: first, and of primal importance, is, that in a very large proportion of cases, some disturbance of sensation will be found, either hyperesthesia or anesthesia, or peculiar sensation. These may be distant from the location of the disease complained of. For example, recently in the female ward of the General Hospital, I was shown a patient who presented the symptoms of general chorea. Suspecting from the character of the movements that they were largely hysterical, I put my finger on her right eye and found the conjunctiva almost completely anesthetic.

A patient sent me by Dr. Park, with rythmical movements of the entire right upper extremity and contraction of the flexor muscles of the hand, had anesthesia from the elbow down, also a narrowing of the visual field of both eyes. This condition developed in a night, during convalescence from a sharp attack of quinsy. The patient had been a heavy drinker for some years. This man was 24 years old, a farmer by occupation, and was not one in whom hysteria would be expected. He was treated by static electricity and spinal douche. He improved rapidly for three weeks, when he found the temptations of the city too great for him, and commenced to drink. The anesthesia, which had disappeared from the arm and back of the hand and three of the fingers, returned one night, and involved the entire hand and wrist. He disappeared one day, and I have lost track of him.

Another case of interest is that of a business man, aged 39, who was engaged to be married. Within four weeks of the date set, he was apparently in perfect health, when suddenly, and without warning, he became aphonic, and, dreading paralysis, he became melancholic.

Examination detected no change of sensation of the skin; fields of vision, normal; smell, and taste, and hearing, normal; but found anesthesia of the pharynx. In addition to the hysteria, he was constipated, and had a heavily-furred tongue. He was treated with cathartics and spinal douches, and in one week he recovered.

A patient sent me by Dr. Boardman presents the following history: Widow, colored, hairdresser; has been afflicted with incomplete loss of power of entire left side for three months. Examination shows diminished sensation of that side, and also sensitive left breast and left ovaralgia.

In five weeks after treatment, the patient was so far recovered as to resume her occupation; the sensory symptoms had entirely disappeared, and the dynamometer showed an increase of twenty pounds in the grip of left hand.

The brevity of this paper should be explained to the society. I desired to limit my report of cases to such patients as I had seen in Buffalo.

388 Franklin Street.

---

A lady who never failed to have her little jest with the doctor all through a painful illness, exclaimed one day when he was announced: "Tell him I'm very sorry, but I don't feel well enough to see him to-day."—*Medical and Surgical Reporter.*

